# Remarkable Case of Phlegmasia Dolens, with Dissection

**Published:** 1846-02

**Authors:** 

**Affiliations:** Physician to the Maternity, Bourdeaux


*Remarkable Case of Phlegmasia Dolens, with Dissection.* By M. Barnetche, Physician to the Maternity, Bourdeaux.—Isab. Bruquet was delivered of her second child on the 2d December, 1844. The duration of the labor was twelve hours, the child presenting in the first position. From the fifth month of pregnancy she had suffered from pains, various in degree, and which continued till the period of delivery. Fifty hours after delivery she had slight milk fever, and on the fourth day she suffered severely from intense pain of the right leg and thigh; when seen at six o'clock that evening by Dr. B., he found the limb swollen, boggy, and warm, and in the whole length of its anterior surface, covered with a violet red line about 2 centimetres (9 lines) broad; enlarged glands were observed under the skin, more especially in the leg; the other portions of the limb were of a dull white colour, and as it were infiltrated: there was also fever. (Forty leeches to be applied on each side, and above and below the red line, linseed meal poultice, and decoction of dog's grass.) On the 7th, pain and inflamation less, redness almost gone, but bogginess continued; lochia natural, slight fever; no pain of abdomen. (Decoction of marsh mallow, warm bath, poultice to whole of limb.) The melioration continued to the 9th, when there was a marked increase of swelling, without any additional pain; complete apyrexia. On the 10th the right leg and thigh had considerable increased in size; and towards the middle of the internal portion of the former, a red spot was discovered; constipation, pulse quick. (Twelve leeches round the inflamed spot, poultice, and a dose of castor oil.) During the night a large phlyctena formed over the spot, which burst, and gave vent to a reddish serosity; no appreciable fluctuation. On the 11th the pulse was full and frequent, the skin dry, tongue red at the point, abdomen soft; abundant exudation of bloody serosity from the phlyctena; deep infiltration of limb, which was very large, and pitted every where on pressure. The left limb now also commenced to be painful, swollen, and of a deep red at the middle portion of its internal surface; six stools, lochia purulent. (Marsh-mallow, wine poultice to the right limb; twelve leeches round the red portion of the left.) Towards evening, the left arm became painful, and the patient also complained of some tenderness over the region of the sacrum, the skin covering which was of the color of the lees of wine. On the 12th, pulse tolerably quiet; complete inability to move the lower extremities; state of arm, tongue, and abdomen the same; no pain on pressure over the pubes. (Wine and water; acidulated decoction of quina; wine poultices to the legs; flannel steeped in camphor to the arm; sacrum to be dusted with the powder of quina and camphor.) On the 14th, there was a marked look of stupidity; the state of the limbs continued the same till the 18th. At that period, the arm ceased to be painful; the poultice was covered with pus; a free issue was given to it by incision; the eschar covering the sacrum had not increased in size, and had become as it were tanned; tongue red, great thirst, pulse febrile. The patient experienced great difficulty in raising the left eye-lid, and in the course of two hours it became altogether impossible to do so. In the course of the evening, the lid of the right eye became affected in a similar way, and it was thought there was some diminution in the power of vision. At his evening visit, Dr. B. found the patient was quite insensible to the light of a candle moved as near the globe of the eye as possible. (Lemonade; acidulated decoction of quina; dressing *ad usum*; soup with wine; blister to the nape of the neck.) 18th. A tolerable night; appearance better; considerable œdema both of the mucous membrane of the palpebræ and eye protruding between the free edges of the former in the left side; on the globe of the eye it was confined to the transparent cornea, now become dull, around which it projected to the extent of a line; commencing œdema of the free edges of the palpebræ of the right side. The left leg had regained its natural size; abundant suppuration from the right leg. (*Ut supra;* blister dressing; compression of the right leg.) 19th. Pulse febrile; mucous membrane of the palpebræ projecting, strangulated here and there, and from two or three points a yellow serosity escaped. 20th and 21st. Little change in the state of the patient; some appetite however. (Same treatment; soups.) During the night of the 21st, the face became flushed and the pulse accelerated. At the morning visit on the 22d, there was fever, with great irregularity in the pulsations of the heart; respiration laborious; crepitating rattle at the summit of the right lung, also in the left, and more marked, the symptoms continued to increase. On the 23, pulse thready; blisters and sinapisms caused no redness of skin; died in the afternoon.

Dissection. *External appearance.*—The body, opened eighteen hours after death, exhaled a strong fetid odour of animal matter in a state of decomposition. The inferior extremities were much swollen and infiltrated. There was gangrenous ulceration over the sacrum; the bone was of a dark grey colour, and filled with pus to the depth of two centimetres. On dissecting the cutis from the right limb, the cellular tissue was found attenuated, of a greyish colour, soaked in liquid, and dissolved as it were in serum. The popliteal and inguinal glands were enlarged and hard, of a whitish-grey colour, and several contained pus; the mesenteric glands were likewise found enlarged, some in a state of suppuration. The left limb was merely œdematous; no pus was found in the cellular tissue, nor were the lymphatic glands enlarged There was evident hardness and redness of the right axillary glands.

*Cranium.*—The brain, cerebellum, medulla oblongata, corpora striata, and thalami, were all in a normal state. There was no serum in the ventricles, but a small quantity of a citron yellow fluid was found between the pia mater and arachnoid, streaked here and there with blood, and small quantities of a fluid pus. The optic nerve, on emerging from the thalami, exhibited no change of colour, but its density was remarakble, creaking under the scissors; there was no infiltration of serum in its sheath. The mucous membrane of the palpebræ and globe of the eye had shrunk; it was of a yellow colour, and the fluid which exuded appeared to be contained in separate cells, but communicating with each other; several layers were removed by means of scissors. The globe of the eye was hard, and with difficulty compressed; a dirty yellow colour was reflected through the transparent cornea, now become dull; on opening it with caution, purulent matter escaped from the anterior chamber, the vitreous humour being replaced by a considerable quantity of greenish pus; neither the colour nor transparency of the lens had undergone any alteration; the same may be said of the fatty cushion on which the globe of the eye rests.

*Chest.*—Two cavities, of the size of a centimetre (five lines) each, were found in the superior lobe of the right lung; they contained pure pus; at a short distance from these, were two globular bodies, of a cretaceous nature, something like tubercle; there was well-marked engorgements of the posterior part of this lobe; as a whole, it was of a deep red colour, and when cut into slices it sunk in water. The inferior lobe of the left lung was almost completely hepatised. There was a small quantity of serum in the left pleura. The heart and pericardium were natural.

*Abdomen.*—The peritoneal coat of the intestines was slightly thickened, especially towards the lower end of the ileum, and over the cœcum, which was much injected. Several red spots were scattered here and there, and in the points corresponding to these, the mucous membrane was soft and easily detached, leaving the walls of the intestines very thin and transparent. The stomach, kidneys, and bladder were natural. Spleen somewhat enlarged, and of a deep red colour; over its surface were scattered patches of a greyish tint; it was soft, and easily broke down under the fingers. Liver large and very dense. On the superior surface of the right lobe, a deep brown patch was discovered, under which there lay a clot of blood, whose diameter in all directions was about half an inch. No other alteration was perceived in the organ.

*Uterus.*—The uterus was of the normal size, but soft to the touch. The ovarian tubes were red; the ovaries themselves tumefied, and softened, and covered with a layer of pus, but without any notable alteration in their substance. On examining the interior of the uterus, its surface was found coated with a species of pap, consisting of decomposed blood and pus. This was also found in several of the veins or sinuses. The tissue of the uterus had lost all its consistency, and by moderate pressure, gave forth a sanious serosity.

*Articulations.*—The pubic and sacro-iliac articulations were healthy.

*Circulatory apparatus.*—Heart and arteries normal.

*Veins.*—A large clot of blood, found in the right auricle, extended upwards in the vena cava superior, and downwards into the vena cava inferior. Its total length was about 16 centimetres (5 inches), a second clot was found in the inferior cava, which extended into the primitive iliac veins, and produced obliteration in the latter, to the extent of about a foot (30 centimetres.) Another clot was found in the right popliteal vein, containing a thick layer of pus in its superior portion; these clots obstructed the calibre of the veins, and, in some points, adhered to them. The internal membrane was, as it were, detached, and adhered to the cloth employed to wipe the surface. The veins of the left limb appeared healthy, as did also those going to the globe of the eye.

Remarks.—Notwithstanding the many works on *phlegmasia alba dolens*, it is a disease with which we are still little acquainted. For ages it has attacked females shortly after delivery, but in degrees so various, and with characters so little marked, that in turn it has been regarded as phlegmon, erysipelas, abscess, rheumatism, metastasis of the milk, &c. By some it has been regarded as rare; thus Hull only witnessed it four times in eight thousand women; whilst M. Velpeau, on the other hand, has met with it five times in eighty deliveries. We ourselves have observed it in still greater proportion; but in some of our cases its presence might have been denied, and it might only have been regarded as a rheumatic affection, or a phlegmonous erysipelas. Be this as it may, we now turn to the morbid appearances discovered, on dissection, in the above case. There are some constitutions which we may term *puogenic*. What are the appearances which reveal the presence of this unhappy organization? Of these we are ignorant and cannot therefore argue as to their being the cause of the disease with which Boquet was attacked. We may remark, however, that during a great part of her pregnancy she suffered severely from irritation, which, from her description, we attribute to a state of sub-acute inflammation of the uterus. Labour only lasted twelve hours, and was unaccompanied with those violent contractions, which are regarded as a cause of contusion of the uterus and soft parts of the pelvis: it could not be accomplished, however, without some pressure on the pelvic vessels and nerves. We may further remark, that the child presented by the first cranial position, and that it was in the right limb, or opposite side, that the disease commenced. This fact is in opposition to the opinion published by M. Velpeau to explain the predilection of phlegmasia dolens for the left limb, viz. the greater frequency of the left occipito-frontal position.

The patient was guilty of no kind of imprudence; but, even supposing that this were not the case, I should still be inclined to regard some unknown predisposition as the most powerful cause of the disease. The symphysis pubis was intact, and exhibited not the slightest alteration in its tissue; the same may be said of the sacro-iliac articulations; we cannot, therefore, attribute the development of the disease to inflammation of these parts. No more can we consider the ulceration of the soft parts over the sacrum, nor the alteration of the periosteum of that bone, as one of its primary causes, as these did not appear till after the disease had made serious progress. Moreover, no pain was felt, either in the symphysis or sacrum, except at the period when the skin was approaching a state of sphacelus. There was no mistaking, during life, the presence of inflammation, both in the glands of the limb and mesentery, and on dissection, we had the most convincing proofs of it. We may therefore infer, that the lymphatic system played an important part in the disease, but the question still remains, was it the first to become inflamed? We cannot deny that pus was found in the large veins; clots of blood apparently obstructed the venæ cavæ, and the iliac veins and their branches of the left side; pus was also found in the uterine sinuses, upon the ovaries and in the lungs; in the latter, indeed, two abscesses were discovered; and at a little distance from these in the same lobe, M. Faure discovered a species of concretion, which he regarded as incipient tuberculisation, but which I am rather inclined to associate with those facts related by Dance in the *Archives Gènerales de Mèdecine* ; in a few days more, this cretaceous matter would probably have become softened, and thus passed into the third stage, in the formation of abscesses in the lungs in cases of uterine phlebitis. Was this pus a consequence of lymphitis? Those who regard the glands as offering a barrier to the passage of pus will reject this idea. Was the presence of pus in the veins an irrefragable proof of phlebitis, and are we to regard inflammation of the veins of the uterus as the only source of the disease which carried off the patient? Another remarkable fact was the loss of vision, which came on so gradually, that we had to take means to satisfy ourselves of its actual extinction. Had we not reason to believe in the existence of some serious affection of that portion of the brain which presides over that function? From its dissection, however, and notwithstanding the coriaceous texture of the optic nerve, may we not affirm that the primary cause was the conversion of the humours of the eye into pus? In fine, our conclusions from the above case are the following: 1st, That all the morbid symptoms observed in the latter months of pregnancy may be referred as well to irritation of the lymphatics as to that of the veins; 2d, That the seat and progress of the inflammation prove that the lymphatic glands were the first attacked; 3d, That the pus formed at first in them was thence carried into the venous circulation, and the remarkable appearances presented by the veins may be regarded as secondary; 4th, That *phlegmasia dolens* is sometimes a phlebitis, and that even when the symphyses may be regarded as the starting points, the general purulent infection always proceeds from the two combined; 5th, Lastly, that the part which the lymphatics play in the production of those phenomena, which, when grouped together, bear the name of *phlegmasia dolens*, is by no means of so secondary a character as has been affirmed in these latter times."--*Journ. de Mèd. de Bordeaux*, as quoted in *Annales de Thèrapeutique*, 7th October 1845.

